# Biosorption of Hg(II) and Cu(II) by biomass of dried *Sargassum fusiforme* in aquatic solution

**DOI:** 10.1186/s40201-015-0180-4

**Published:** 2015-03-17

**Authors:** Shengmou Huang, Gan Lin

**Affiliations:** School of chemical engineering and food science, Hubei University of Arts and Science, Xiangyang, 441053 China

**Keywords:** Biosorption, *Sargassum fusiforme*, Mercury, Copper

## Abstract

The biosorption of heavy metals Hg(II) and Cu(II) from aquatic solution by biomass of dried *Sargassum fusiforme* was studied in the paper. The *Sargassum fusiforme* was able to absorb appreciable amount of mercury and copper from the aquatic solutions within 60 min of contact time with the metal solution and exhibited high removal of mercury and copper at low equilibrium concentrations. The specific adsorption of both Hg(II) and Cu(II) increased at low concentration of biomass and decreased when biomass concentration exceeded 2.0 g/L. The binding of mercury followed Freundlich model while copper supported Langmuir isotherm for adsorption with their r^2^ values of 0.971 and 0.923, respectively. The maximum adsorption per unit masses of *Sargassum fusiforme* (mg/L) at equilibrium (q_max_) for Hg(II) and Cu(II) were calculated to be 30.86 and 7.69 mg/g, respectively. The biosorption by *Sargassum fusiforme* was best described using a pseudo-second-order kinetic model for copper and mercury ions in solution in the study. The adsorption was pH dependent as the maximum mercury biosorption and copper adsorption was happened at solution pH of 8–10.

## Background

The heavy metal is among the most common pollutant found in industrial effluents. The major sources of pollution in the aquatic environment are industries such as paint, pulp and paper, oil refining, electrical, rubber, processing, fertilizer, pharmaceutical and battery manufacturing [1,2]. The major effects of mercury and copper poisoning manifest as neurological and renal disturbances as it can easily pass the blood–brain barrier. Environmental contamination with toxic heavy metals is a significant worldwide problem with their successive accumulation in the food chain and continued persistence in the ecosystem which will hurt human beings. Efforts have been made to remove toxic heavy metal from the wastewater and environment by using conserved technologies such as ion exchange or chemical precipitation, which are sometimes inefficient and expensive, particularly for removal of low concentrated heavy metal ions [3], and also leads to produce toxic sludge that adverse the economical feasibility of the treatment methods.

Indeed, many early studies have shown that nonliving biomass may be even more effective than living cells in sequestering metallic elements. Over the past two decades, much effort has been directed at identifying readily available biomass which, in its nonliving state, is capable of effectively removing heavy metals. It has been demonstrated that biosorption is a potential alternative to traditional treatment processes of metal ions removal. Biosorption is a property of certain types of inactive, dead biomass to bind and concentrate heavy metals from even very dilute aqueous solutions [4]. Biomass exhibits this property, acting just as a chemical substance, as an ion exchange of biological origin. Research on biosorption is revealing that it is sometimes a complex phenomenon where the metallic species could be deposited in the solid biosorbent through different sorption processes of ion exchange, complexation, chelation, microprecipitation, etc.

In these years, the role of seagrass in removal of toxic metal ions from the polluted water have taken more importance as they have a high area-to-volume ratio and therefore provide a large contact area for metal binding [5]. Small bacteria, algae, fungi and yeast have been well recognized for heavy metal removal [6]. Up to date, the role of algae has received increased attention to years because of it are potential for application of environmental protection as well as recovery of heavy metals. The affinity with various algae species for binding of heavy metals shows different results [7]. Metal ions with greater electro-negativity and smaller ionic radii are preferably absorbed by algae biomass [8]. Metal accumulation capacity of algae biomass is sometimes higher than chemical sorbents therefore algae biomass may serve as an economically feasible to the existing physicochemical methods of metal removal of wastewaters. The major challenge to biosorption studies is to select the most promising biomass from a large pool of available and inexpensive biomaterials. Contributions to *Sargassum fusiforme* in the metal adsorption is of great concern [9].

In the present study, dried biomass of *Sargassum fusiforme* were used and characterized for its Cu(II) and Hg(II) removal potential from synthetic metal solution. The effect of pH, biomass concentration, initial metal concentration and contact time was studied for metal sorption procedure as well as heavy metal equilibrium sorption kinetics was made in lab.

## Methods

### Biosorbent material

The dry biomass of *Sargassum fusiforme* were purchased from Fuzhou Lantian limited Company, Fujian (China). It was powdered and sieved into less than 1 mm in diameter and dried at 80°C for 12 h with drying oven(01A, Suzhou sinovel oven manufacturing co., LTD). The characteristics of the *Sargassum fusiforme* were determined by F-Sorb type 3400 specific surfaces and pore diameter gauge(Beijing gold spectrum technology co., LTD). The surface BET on the algae was of means with 35-40 m^2^/g, total pore volume is of 50-70% and the pore structure is mesoporous material. The FTIR spectrum of the fresh and metal-loaded ones were made by the FTIR920(Tianjin topology instrument co., LTD). The SEM images of the fresh and metal-loaded ones were attained by SU3500(Hitachi High-Technologies Corporation).

### Mercury and copper sorption experiments

Heavy metal solutions were prepared for diluting 200 mg/L of stock solutions, which were made by dissolving copper nitrate and mercury nitrate of analytical grade (Shanghai bo yiu biological technology co., LTD) in double distilled water. Metal sorption studies were carried out to evaluate the capacity of dry biomass of *Sargassum fusiforme* to adsorb metal ions from solutions. In batch ones, 100 mL of synthetic metal solutions having different concentrations (10,20,30,40, and 50 mg/L) of copper or mercury were placed in 250 ml Erlenmeyer flasks with a range of biomass concentrations(0.5,1.0,1.5.2.0,2.5, and 3.0 g/L) as biosorbent. Erlenmeyer flasks were kept under shaking at 120 rpm at 25°C. Samples were taken after 20-120 min and filtered and analyzed for final metal concentration (C_f_) using ICP-AES (HK-2000, Huake Beijing tiancheng technology co., LTD) after an acid digestion [10].

To see the effect of pH on Hg(II) and Cu(II) removal, a range of pH (2–10) was adjusted to 0.1 M NaOH or 0.1 M HCl in 100 ml metal solutions containing fixed concentration of metal at 10 mg/L and biomass of 3.0 g/L followed by contact time of 60 min at rotation of 120 rpm. The metal adsorption(q) with *Sargassum fusiforme* and bioremoval efficiency(R) were calculated by the following formulae.

1 $$ q=\left({C}_i-{C}_f\right)\ast \frac{V}{M} $$

2 $$ R\left(\%\right)=\frac{C_i-{C}_f}{C_i}\ast 100 $$

Where q = metal adsorption (mg/g); M = dry mass of *Sargassum fusiforme*(g); V = volume of initial metal solution used (L); R = bioremoval efficiency (%); C_i_ = initial concentration of metal in aquatic solution (mg/L); C_f_ = final concentration of metal in aquatic solution (mg/L) [11].

### Adsorption isotherm

During biosorption, the equilibrium is established between absorbed metal ion on the *Sargassum fusiforme*(q) and unabsorbed metal ions in the solution(C_f_). This equilibrium represented by Langmuir and Freundlich adsorption isotherms, are widely used to analyze data for wastewater treatment application [12]. Langmuir equation, which is valid for monolayer sorption onto a surface, with identical sites was given by Eq. 3.

3 $$ q={q}_{\max}\ast b\ast \frac{C_f}{1+b{C}_f} $$

Where q_max_(mg/g) is the maximum amount of the metal ion per unit weights of algae to form a complete monolayer on the surface bound at high C_f_(mg/L), and b is a constant related to the affinity of the binding sites(mg/L), q_max_ represents a practical limiting adsorption capacity when the surface is fully covered with metal ions and assists in the comparison of adsorption performance [13]. The q_max_ and b can be determined from the liner plot of C_f_/q versus C_f_. The empirical Freundlich equation based on sorption on a heterogeneous surface is given below by Eq. 4.

4 $$ q=k\ast {C}_f^{1/n} $$

The k and n parameters are the constants of the Freundlich isotherm. The k and n are indicators of adsorption capacity and adsorption intensity, respectively. The Eq.4 can be linearized in logarithmic forms and Freundlich constants can be determined by the plot. Freundlich isotherm is also more widely used as it provides no information on the monolayer adsorption capacity [14].

### Biosorption kinetics

The experimental biosorption kinetic data was modeled using the pseudo-first-order (Eq.5), and pseudo-second-order models (Eq.6). The linear pseudo-first-order model can be represented by the following equation:

5 $$ \log \left({q}_e-{q}_t\right) \log {q}_e-\frac{K_1}{2.303}\ast t $$

The qe (mg/g) and qt (mg/g) are the amounts of adsorbed metal on the sorbent at the equilibrium time and at any time t, respectively, and K_1_(min^−1^) is the rate constant of the pseudo-first-order adsorption process. The linear pseudo-second-order model can be represented by the following equation:

6 $$ t/{q}_t=\frac{1}{K_2}\ast {q}_e^2+\frac{t}{q_e} $$

Where K_2_ (g*mg^−1^*min^−1^) is the equilibrium rate constant of pseudo-second-order [11].

## Results and discussion

### Effect of pH solution

**Figure 1:**
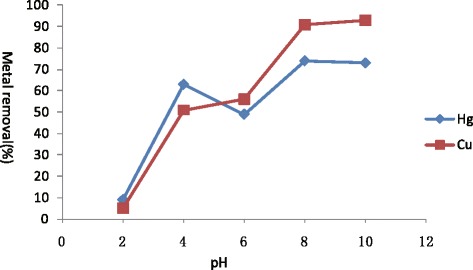
**pH dependent sorption of Hg(II) and Cu(II) by** ***Sargassum fusiforme*** **(Hg(II):10 mg/L, Cu(II):10 mg/L), standard deviation is less than 5% in triplicates.**

The pH is one of the important parameters in heavy metal sorption by *Sargassum fusiforme.* Therefore metal sorption studies were carried out at different pH values. Results revealed the maximum biosorption of Hg(II) were at pH 8(70%) and 10(72%) and Cu(II) demonstrated the same result at pH8(90%) and 10(92%) from aquatic solution containing initial 10 mg/L of metal concentration (Figure 1).

It was observed that Hg(II) and Cu(II) adsorption was less than 10% at pH 2. Enhance adsorption of Hg(II) and Cu(II) ions at higher pH was observed which coincides with earlier findings where in most cases the removal efficiency increased steadily with rise in pH [15]. The adsorption of metal ions was lower at low pH because of high concentration of protons in the solution which competed with metal ions in forming a bond between the active sites on the surface of the algae biomass [16]. Selective sorption of specific metals due to distinct pH optima for their sorption may be due to the chemical composition of cell surfaces. A distinct relationship between pH of aquatic metal solutions and involvement of functional group in binding of Hg(II) and Cu(II) onto *Sargassum fusiforme* maxima was observed with the involvement of functional groups such as carboxyl, phosphate and hydroxyl [17].

### Metal concentration

**Figure 2:**
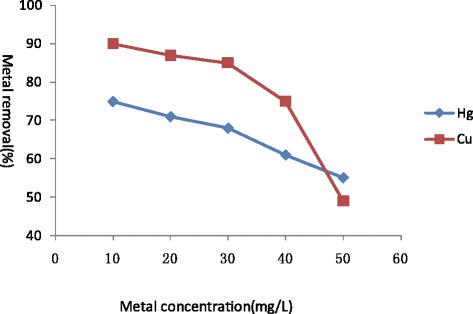
**Initial concentration dependent sorption of Hg(II) and Cu(II) by** ***Sargassum fusiforme*** **, standard deviation is less than 5% in triplicates.**

Biosorption studies carried out for both Hg(II)and Cu(II) in 100 ml solutions containing metals varying from 10 to 50 mg/L with Hg(II)and Cu(II) by 3.0 g/L of biomass exhibited effective role of initial metal concentration on metal removal. A consistent decrease in metal removal was observed by increasing external metal concentration (Figure 2) where 70-74% removal efficiency was reported on 10 to 20 mg/L Hg(II) solutions followed by decline in solutions to 50 mg/L Hg(II)(Figure 2). Similarly decreases in Cu(II) removal efficiency was observed by increasing external metal concentration on 50 mg/L Cu(II)(Figure 2). Rapid metal adsorption profile of *Sargassum fusiforme* was obtained for both Hg(II) and Cu(II), which is important when the algae are used for biosorption. It exhibited rapid biosorption at first 60 min by removing 72% Hg(II) and 90% of Cu(II) from metal solutions. Decreases in metal removal of increasing initial metal concentration was supported by the findings that observed that the removal of metal generally decreases from increasing concentration of metals in the solution [18]. Algae surface has different functional groups of varying the affinity with ionic kinds, therefore decline in metal removal is largely attributed to saturation of adsorption sites [7].

### Contact time determination

**Figure 3:**
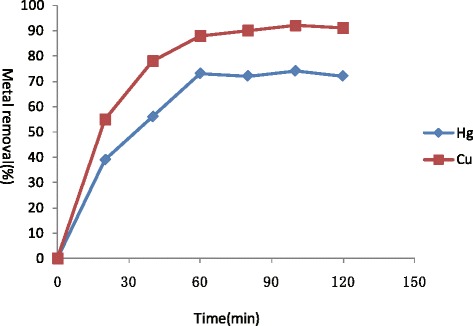
**Time dependent removal of Hg(II) and Cu(II) by** ***Sargassum fusiforme*** **(Hg(II):10 mg/L, Cu(II):10 mg/L), standard deviation is less than 5% in triplicates.**

Rapid metal adsorption profile of *Sargassum fusiforme* was obtained for both Hg(II) and Cu(II), which is important when the material is to be used for biosorption. It exhibited rapid biosorption in first 60 min by removing 71% Hg(II) and 88% of Cu(II) from metal solutions thereafter increase in metal removal was magical. Equilibrium was established between absorbed metal ions after 40 min with maximum removal of 55% and 75% of Hg(II) and Cu(II) (Figure 3).

The *Sargassum fusiforme* biomass showed rapid biosorption at first 60 min. It has been reported that the sorption of heavy metal ions by algae followed mechanism where the metal ion is physically or chemically taken up onto the surface of the algae [19]. In this case, since the algae were dried and biological functions were no longer active, the sorption could only take place on the cell surface. The increase in Hg(II) and Cu(II) adsorbed by increasing biomass was also expected as a result of increase in available area-to-volume ratio and therefore providing a large contact area for heavy metal binding [20].

### Biomass determination

**Figure 4:**
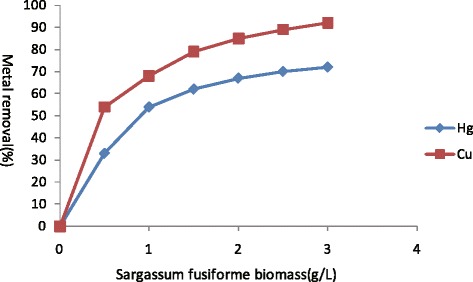
**Effect of** ***Sargassum fusiforme*** **biomass concentration on Hg(II) and Cu(II) removal(Hg(II):10 mg/L, Cu(II):10 mg/L), standard deviation is less than 5% in triplicates.**

Increase in metal removal efficiency from 10 mg/L manual metal solutions to both Hg(II) and Cu(II) was observed on increasing biomass concentration (Figure 4).

Hg(II) adsorption was increased to 71% by increasing biomass concentration on 0 to 3.0 g/L in 10 mg/L of mercury containing synthetic solutions whereas, same trend was observed in case of Cu(II) with the increase of 90%. The *Sargassum fusiforme* biomass displayed its equilibrium for Hg(II) removal of 3.0 g/L of biomass concentration whereas, a continuous increase in Cu(II) removal of increasing biomass concentration was observed of 3.0 g/L. Metal adsorption studies from solution mass balance revealed a decline in sorption of Hg(II) and Cu(II) respectively by increasing biomass from 0 to 3.0 g/L after 60 min of contact time. The other probable explanations for such a relationship between biomass concentration and adsorption may be limited availability of metal, increased electrostatic interactions between binding sites and reduced mixing at higher biomass concentration [21,22].

### The FTIR and SEM analysis

**Figure 5:**
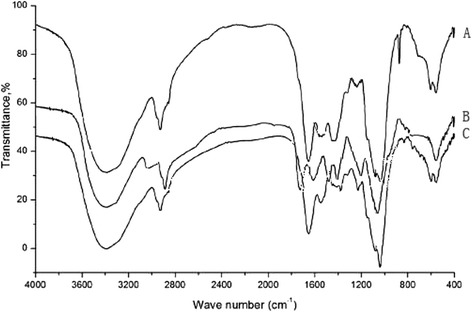
**The FTIR spectrum of the adsorbent for fresh and metal loaded ones.** **A**: fresh, **B**: Cu(II) loaded, **C**: Hg(II) loaded.

**Figure 6:**
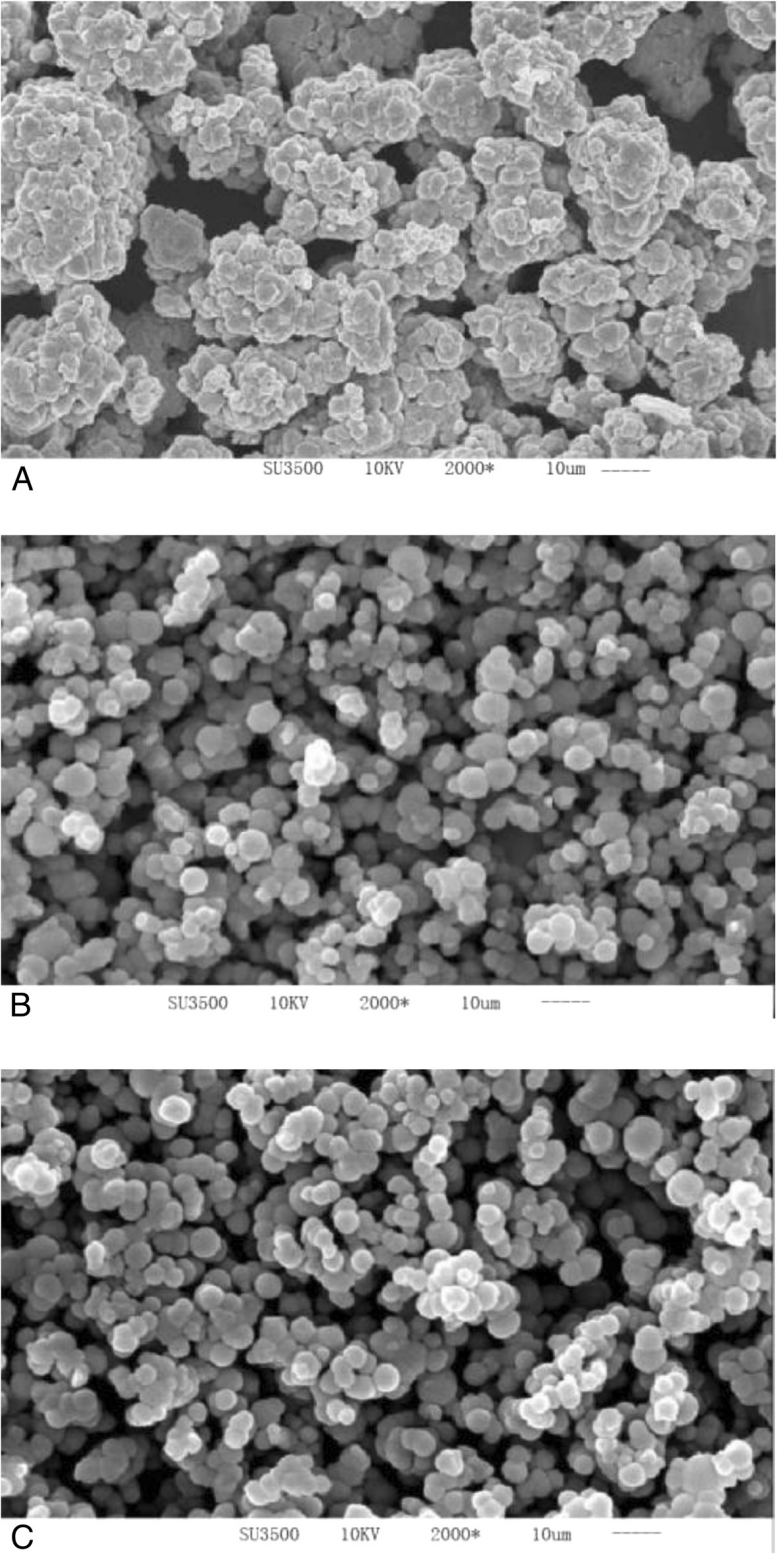
**The SEM images of the adsorbent for fresh and metal loaded ones. A:** fresh**, B:** Cu(II) loaded, **C:** Hg(II) loaded.

With the determination of functional matrices and group in the heavy metal biosorption of the algae, it is useful for the study of FTIR spectrums. The figures are shown in Figure 5. With the determination of biosorption kinetics and isotherms in the heavy metal biosorption of the algae, the use of SEM images is shown in Figure 6 for the study.

### Modeling study

A linear regression of the experimental results from Hg(II) and Cu(II) differed in terms of Hg(II) adsorption fitted better to Freundlich isotherm and Cu(II) to Langmuir isotherms with r^2^ values of 0.971 and 0.823 respectively (Table 1). The maximum adsorption per unit masses of *Sargassum fusiforme* (mg/L), at equilibrium (q_max_) for Hg(II) and Cu(II) were calculated to be 30.86 and 7.69 mg/g respectively (Table 1).

**Table 1:** **Langmuir and Freundlich parameters for the sorption of the test metals by** ***Sargassum fusiforme***

**Metal**	**Langmuir isotherm**			**Freundlich isotherm**		
	**b(L/mg)**	**r** ^**2**^	**qmax(g/mg)**	**K(mg/g)**	**n**	**r** ^**2**^
Hg(II)	0.988	0.668	30.86	2.012	3.89	0.971
Cu(II)	1.269	0.923	7.69	0.899	5.41	0.765

The sorption isotherm is the relationship between equilibrium concentration on the solution and equilibrium concentration of solute in the sorbent at constant temperature where either Freundlich or Langmuir model can describe the biosorption equilibrium of copper and mercury [23]. An extremely high r^2^ value of Freundlich isotherm for Hg(II) sorption indicated that ion exchange interaction takes place between metal ion and the biosorbent, while Cu(II) follows Langmuir isotherm and thus supported physicochemical interactions with each other.

For biomasses of *Sargassum fusiforme,* the kinetics of copper and mercury biosorption were analyzed using pseudo-first-order and pseudo-second-order models. All the constants and regression coefficients are shown in Table 2. In the present study, biosorption by *Sargassum fusiforme* was best described using a pseudo-second-order kinetic models for copper and mercury ions in solution. This adsorption kinetic is typical of the adsorption of divalent metals onto biosorbents.

**Table 2:** **First and second order adsorption rate constants for Hg(II) and Cu(II)**

**Metal**	**Pseudo-first-order**		**Pseudo-second-order**	
	**K**_**1**_ **(min** ^**−1**^ **)**	**r** ^**2**^	**K**_**2**_ (g*mg ^**−1**^ *min ^**−1**^ **)**	**r** ^**2**^
Hg(II)	5.3*10^−3^	0.745	9.15*10^−3^	0.957
Cu(II)	3.1*10^−3^	0.615	8.84*10^−3^	0.963

## Conclusions

The goal of the study was to explore and find out the potential use of algae biomass as a low cost sorbent for the removal of heavy metals from aquatic solutions. The heavy metals Hg(II) and Cu(II) from aquatic solution was able to be dealt with dried *Sargassum fusiforme*. The seagrass adsorbed appreciable amount of mercury and copper from the aquatic solutions within 60 min at low equilibrium concentrations. The specific adsorption of both Hg(II) and Cu(II) increased at low concentration while decreased when biomass concentration exceeded 2.0 g/L. The binding of mercury followed Freundlich model but copper supported Langmuir isotherm for adsorption with their r^2^ values of 0.971 and 0.923, respectively. The maximum adsorption per unit masses of *Sargassum fusiforme* (mg/L), at equilibrium (q_max_) for Hg(II) and Cu(II) were calculated to be 30.86 and 7.69 mg/g respectively. The adsorption was pH dependent as the maximum mercury biosorption utilized at pH 8 and 10 and Cu(II) adsorption was at pH 8 and 10. The biosorption by *Sargassum fusiforme* was best described using a pseudo-second-order kinetic model for copper and mercury ions in solution in the study. The present paper emphasizes the *Sargassum fusiforme* is an ideal candidate and can be designed as a practical and economical process for wastewater treatment polluted by heavy metals.
